# Dynamic Fault Tree Generation and Quantitative Analysis of System Reliability for Embedded Systems Based on SysML Models

**DOI:** 10.3390/s24186021

**Published:** 2024-09-18

**Authors:** Changyong Chu, Weikang Yang, Yajun Chen

**Affiliations:** 1 School of Mechanical Engineering, Hangzhou Dianzi University Information Engineering College, Hangzhou 311305, China; 2 State Key Laboratory of Digital Manufacturing Equipment and Technology, Huazhong University of Science and Technology, Wuhan 430074, China; 3 School of Mechanical Engineering, Hangzhou Dianzi University, Hangzhou 310018, China; 232010126@hdu.edu.cn (W.Y.); 212010046@hdu.edu.cn (Y.C.)

**Keywords:** model-based systems engineering (MBSE), failure modes and effects analysis (FMEA), profile extension, risk assessment, dynamic fault tree, quantitative analysis

## Abstract

As embedded systems become increasingly complex, traditional reliability analysis methods based on text alone are no longer adequate for meeting the requirements of rapid and accurate quantitative analysis of system reliability. This article proposes a method for automatically generating and quantitatively analyzing dynamic fault trees based on an improved system model with consideration for temporal characteristics and redundancy. Firstly, an “anti-semantic” approach is employed to automatically explore the generation of fault modes and effects analysis (FMEA) from SysML models. The evaluation results are used to promptly modify the system design to meet requirements. Secondly, the Profile extension mechanism is used to expand the SysML block definition diagram, enabling it to describe fault semantics. This is combined with SysML activity diagrams to generate dynamic fault trees using traversal algorithms. Subsequently, parametric diagrams are employed to represent the operational rules of logic gates in the fault tree. The quantitative analysis of dynamic fault trees based on probabilistic models is conducted within the internal block diagram of SysML. Finally, through the design and simulation of the power battery management system, the failure probability of the top event was obtained to be 0.11981. This verifies that the design of the battery management system meets safety requirements and demonstrates the feasibility of the method.

## 1. Introduction

With the continuous development of computer and electronic technology, embedded systems are integrating increasingly complex functions, leading to a corresponding increase in system complexity [1,2]. To assess the potential risks of such complex systems, fault mode and effects analysis (FMEA) or fault tree analysis (FTA) are often used to analyze system security [3,4,5,6,7,8]. However, traditional safety and reliability analysis methods such as FMEA and FTA are often disconnected from system design, which means safety engineers must use other tools for analysis. This leads to significant costs associated with correcting errors discovered during later safety analyses, which are often several times higher than in the early stages of system design [9,10,11]. To address these limitations, Model-Based System Engineering (MBSE) [12,13,14] technology has emerged as an increasingly important means of ensuring security and reliability. Compared with traditional document analysis, modeling offers greater expressive power [15]. However, there is often a gap between the design process and security analysis, and security analysis results are not updated as system design changes [16]. Therefore, integrating security and reliability analysis techniques with MBSE model design represents a new path forward.

In this paper, we propose a method for integrating security and reliability analysis into systems engineering. Our approach leverages the block definition diagram and activity diagram of SysML model to automatically generate an FMEA table. Using the feedback from the FMEA safety analysis results, the system design is modified in a timely manner to meet safety performance requirements. A safety profile is then established to extend the SysML model, and a dynamic fault tree is automatically generated using a traversal algorithm. The logic gate of the dynamic fault tree is described by a parametric diagram. Finally, we perform a quantitative analysis of the dynamic fault tree to evaluate the probability of failure for top-level events.

The paper is structured as follows: Section 2 provides an overview of related work on fault tree generation and compares it with our method. Section 3 outlines our approach for generating FMEA from the system model, improving the system design based on safety evaluation results, and automatically generating dynamic fault trees from the improved model. Then, we introduce the use of SysML parametric diagrams to represent dynamic fault tree logic gates and perform quantitative analysis based on probability using the modeling tool. Section 4 presents a case study of a power battery management system to demonstrate the feasibility of our approach. Finally, we draw conclusions in Section 5.

## 2. Related Work

This section delves into efforts to integrate security analysis into MBSE, with a particular focus on FMEA generation or fault tree generation, as detailed in references [17,18,19,20]. The analysis of both FMEA tables and dynamic fault tree generation methods can be effectively supported by SysML [17,21,22,23]. SysML is an OMG (Object Management Group) standard utilized to design, analyze, and verify complex systems such as hardware, software, information, personnel, and procedures and to communicate requirements and corresponding designs among engineers. Thus, integrating safety and reliability analysis with MBSE can effectively reduce the occurrence of errors caused by manual reasoning. The integration of safety analysis with Model-Based Systems Engineering (MBSE) has mainly focused on the generation of Failure Modes and Effects Analysis (FMEA) [18,19] or the generation of fault trees [20,24,25].

David et al. [18] constructed a semi-automatic model in AltaRica data flow using SysML models. They studied and compared the SysML language with the AltaRica language and ultimately had safety experts complete the FMEA report. Hecht et al. [19] employed model transformation techniques to convert SysML models representing system structure and fault modes into AltaRica models and subsequently generated FMEA tables from these models. However, these methods rely more on security experts to create the FMEA table manually, and the specific failure reasons need to be added by security experts. This article uses the “anti-function” method to obtain the cause of the component’s failure by taking the corresponding activity as the opposite, reducing human involvement in the automatic generation process.

Xiang J et al. [20] proposed a Reliability Configuration Model (RCM) and a Static Fault Tree Model (SFTM). They converted SysML models into RCM models and embedded the system configuration information required for reliability analysis into the RCM model. Finally, they generated static fault trees based on the RCM specification. Faida Mhenni et al. [24] represented SysML Internal Block Diagrams (IBDs) as directed graphs and divided them into multiple modules. Through graph traversal and pattern recognition algorithms, they automatically derived partial fault trees for corresponding modules with respective logic gates and basic events. These partial fault trees were assembled to form the final fault tree. Yakymets N et al. [25] proposed a methodology and associated framework for the safety assessment of robotic systems in the early phases of development. The method uses model-based fault tree analysis for the initial safety assessment of safety-critical robotic systems, and the proposed framework is capable of automatically or manually constructing fault trees and performing qualitative and quantitative fault tree analysis.

The research efforts mentioned above are focused on various FMEA- or FTA-generating methods. However, they do not carry out further reliability analysis after completing the FMEA or FTA generation. Moreover, only spare gates are considered in the dynamic fault tree generating process, without further development regarding functional dependency gates, priority AND gates, and the like. Aiming at these problems, this paper proposes a method for generating dynamic fault trees considering temporality and redundancy based on an improved system model, followed by quantitative analysis. First, the risk assessment results based on FMEA are used to enhance the system design to meet regulatory requirements. Then, the block definition diagram is expanded to describe module-fault semantics, and in conjunction with activity diagrams, a traversal algorithm is employed to automatically generate dynamic fault trees. Finally, within the SysML internal block diagram, a parametric diagram is created to describe the logical gate operations of the dynamic fault tree for quantitative analysis. Overall, this method provides a more comprehensive and effective approach to risk assessment during the design stage, allowing for a more thorough analysis and better-informed decision-making throughout the entire design process.

## 3. Methodology

### 3.1. Dynamic Fault Trees Generation and Quantitative Analysis Approach

**Figure 1:**
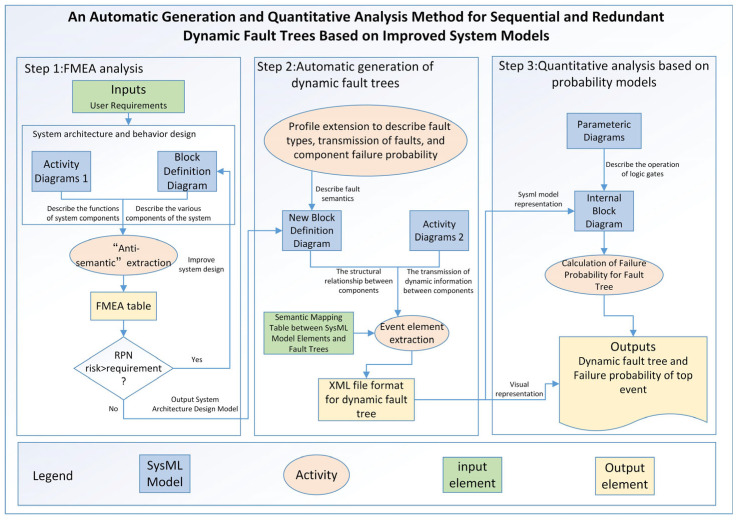
The automatic Dynamic Fault Trees Generation and quantitative analysis approach.

In order to integrate the embedded system safety analysis process into MBSE, ensuring consistency between system design and safety components, this paper proposes a method for automatic generation and quantitative analysis of dynamic fault trees based on an improved system model, with considerations for temporal aspects and redundancy. The specific process is illustrated in Figure 1. Step 1: Initiate an FMEA analysis and establish SysML block definition diagram and activity diagrams based on user requirements. By employing “anti-semantic” approach, the SysML model is traversed to generate an FMEA table. Safety experts then conduct a risk assessment, and components with a risk priority number (RPN) exceeding the specified value require design improvements. Step 2: Utilizing profile extension mechanisms, extend the block definition diagram to enable the description of fault types, fault propagation, and component failure probabilities. Create a semantic mapping table between SysML elements and dynamic fault trees. An algorithm is employed to automatically extract event elements and generate dynamic fault trees. Step 3: Employ SysML parametric diagrams to represent the operational rules of fault tree logic gates. Establish internal block diagrams to represent dynamic fault trees, facilitating the subsequent calculation of top-level event failure probabilities within the fault tree.

### 3.2. FMEA Analysis Process

This section describes the process of integrating FMEA with MBSE. FMEA is a widely used reliability tool for safety analysis. Its purpose is to identify potential failures that could cause system function failure. It enables easy and cost-effective modifications to the system design process errors, thereby reducing the maintenance costs of the system in the future. This section provides methodological steps for integrating FMEA analysis into systems engineering.

Step 1: Establish SysML block definition diagrams and activity diagrams to define the system requirements that describe the system’s functionality. The block definition diagram represents system functional components, while the activity diagram describes the interactions between components.

Step 2: Functional Architecture Definition: In this step, the main functions of the system are decomposed into a hierarchical model of sub-functions. In SysML, system functions are represented by activities and functional analysis is modeled using multiple activity diagrams. Each activity diagram represents a process where a function is decomposed into sub-functions, and each activity diagram displays the transformation from input to output flows.

Step 3: Logical Architecture Definition: The previous step (Step 2) defined a functional architecture that forms a logical architecture by assigning functions to system components.

Step 4: Risk Function Assessment: This step involves automatically generating the FMEA analysis table from the SysML model built in the previous two steps. The FMEA table is used to identify faults and analyze potential hazards caused by those faults. Column headings mainly include component names, functions, failure modes, potential causes, risk priority numbers, etc. The automatic generation of FMEA is achieved by extracting information from XML files using an algorithm [26,27]. Extract system element nodes, which represent the component names, from the XML file corresponding to the block definition diagram. Information about component functions and failure modes is typically represented using activity diagrams. The name of the activity diagram represents the function of the component, allowing the failure modes to be generated using a “reverse function” approach. For example, if the function of a component is to transmit information, its failure mode could be “information transmission failure”. The activities in the activity diagram represent the reasons for the failure of the function, which can be obtained by taking the opposite meaning of the activity description.

Step 5: After completing the hazard identification analysis on the FMEA table, it is necessary to conduct a risk assessment. Safety experts use existing data to expand the FMEA, focusing on the severity (S), occurrence (O), and detectability (D) parameters that are missing in the FMEA table [28,29]. Finally, the risk priority number (RPN) of the hazard event is determined by the combined level of these three parameters [30]. In this risk analysis method, the RPN is the dependent variable of the function (F). This can be expressed as Equation (1) [31,32].

RPN = S × O × D(1)

The degree of influence of the three independent variables S, O, and D is measured on a scale of 1–10, with 10 representing the most severe effects and 1 representing the least. After determining the fault execution degree, such as 95%, the RPN is lower than (1–95%) × 10 × 10 × 10, which is 50, indicating that we need to trace and solve faults with an RPN value greater than 50. After obtaining the RPN value from Equation (1), we need to evaluate the severity of each fault mode with a risk priority number exceeding 50. When the severity of the fault is significant, at least one safety requirement should be added. Design changes can start from the early design stage at the functional level to eliminate or reduce identified risks. By modifying elements with higher risk priority numbers and adding new fault tolerance mechanisms, the reliability of the component can be improved [33], and the key fault modes of elements with higher risk priority numbers can be eliminated. This can reduce the effect on the main functions of the system, and the modified fault tolerance mechanism needs to be updated in a timely manner in the SysML model.

### 3.3. Generation of Dynamic Fault Tree

#### 3.3.1. Semantic Expansion of Fault Descriptions

Components with high-risk priority numbers were identified during the risk assessment in Section 3.2, and fault tolerance mechanisms were implemented accordingly. To capture these measures in the SysML model, UML extension mechanisms were leveraged to create security profiles representing the fault tolerance mechanisms. Specifically, the SysML Profile package was extended with elements such as stereotypes, constraints, and tagged values to address modeling needs. New stereotypes were added to describe fault semantics, providing additional semantics or constraints without altering the original model structure and content.

(1) Expanding Redundancy Based on Profile

To reduce the risks associated with high-risk components, we primarily use redundant structures [34,35], which employ spare parts. When the primary component fails, the spare component takes over, and the spare construction can be classified into three types based on the backup method: cold, warm, and hot spares. Cold spares are in hibernation mode while the primary part is working; warm spares are in a state between sleep and wakefulness while the primary parts are functioning and have a certain failure probability of α (0 < α < 1) times the normal operation. Hot spares are in the active state, like the primary parts.

**Figure 2:**
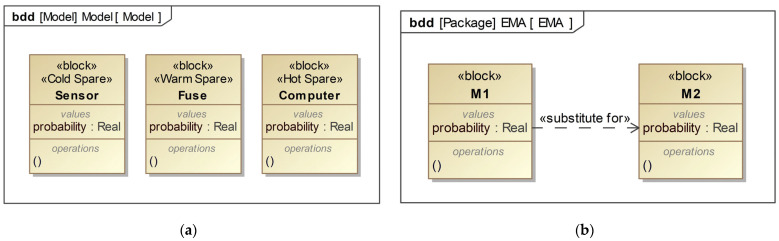
Extension method for stereotype of spare parts and stereotype “Substitutes for”: (**a**) stereotype of spare parts; (**b**) stereotype “Substitutes for”.

The configuration of the Sensor block shown in Figure 2a indicates that the block has a cold spare parts feature. The cold spare part is in a hibernation state and will be activated to work once the main component fails. At the same time, backup data can be imported into the cold spare parts. The Fuse block is a warm backup component that has a certain probability of failure before operation. The computer block has the characteristic of a hot spare part, which works together with the main component. After the main component fails, it will replace the main component to continue working.

For two components with the same functionality, this article uses the stereotype “Substitutes for” to represent them, as shown in Figure 2b. M1 and M2 are two components with the same function. When one component fails, another component with similar function will replace it and continue to work. Only when both components fail will the system fail.

(2) Expanding Timeliness Based on Profile

**Figure 3:**
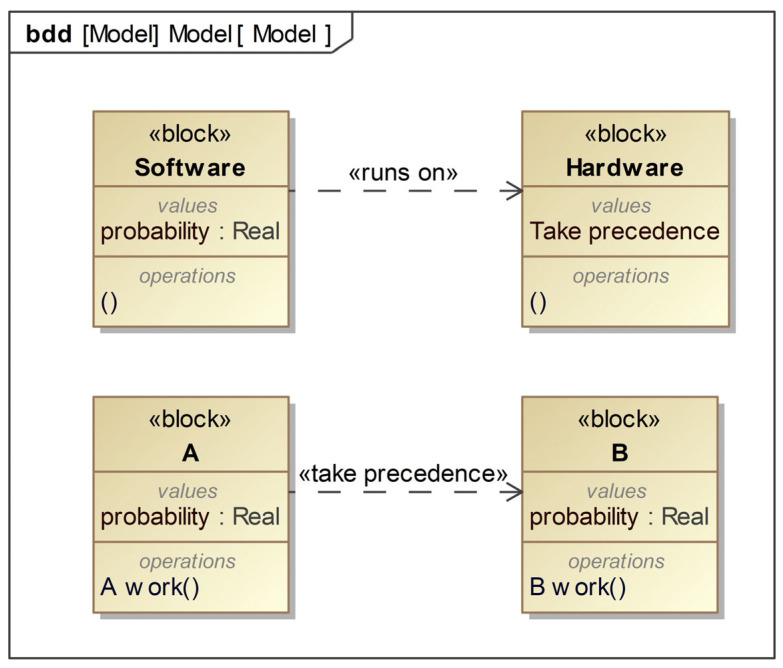
Profile extension for “temporal nature”.

In response to the “temporal” characteristics of dynamic fault trees, this article establishes stereotypes “runs on” and “take precedence”, as shown in Figure 3. They are both dependent on dependency relationships, with the client pointing to the provider.

This means that when the provider element changes, the client element also needs to change. The “runs on” stereotype represents the relationship between software and hardware. Software runs on hardware, and a hardware fault can cause software to fail, but a software fault does not affect hardware. The stereotype “take precedence” indicates a priority relationship between two components, and the system will only fail if A and B fail in order. The order of its failure is determined by the order of actions in the activity diagram. After the system starts working, according to the control flow, A work is first followed by B work, so only if A fails first and if B fails again will the system fail.

The SysML block definition diagram before expansion can only describe the static logical relationship between blocks, while the SysML block definition diagram after expansion can describe the redundant and dependency relationships of block failures and can correspond one-to-one with the logical gates of the dynamic fault tree. Table 1 shows the semantic correspondence between the SysML model and the dynamic fault tree. The SysML block corresponds to the basic event of the dynamic fault tree, which only represents the failure of the corresponding component and is independent of the specific failure mode of the component.

#### 3.3.2. Automatic Generation of Dynamic Fault Trees

The process of fault propagation typically involves faults being transmitted between physical units at different levels, as well as between physical units at the same level. However, this paper only focuses on fault transmission between different physical units. To accomplish this, the physical domain model is broken down into multiple subsystems. These subsystems typically receive fault-related information from their components. Once the top event is determined, the fault tree can be generated using the fault propagation relations and rules. Typically, the fault transmission between upper and lower levels is defined by a logical gate. If the fault cause does not contain the cause of the next level, it is considered a basic event. On the other hand, if the cause of the next level is included, it is classified as an intermediate event.

Therefore, the expanded block definition diagram is limited to representing the fault information of the physical domain and cannot describe how faults propagate between blocks. However, the SysML activity diagram can illustrate the transmission of fault information between blocks.

**Figure 4:**
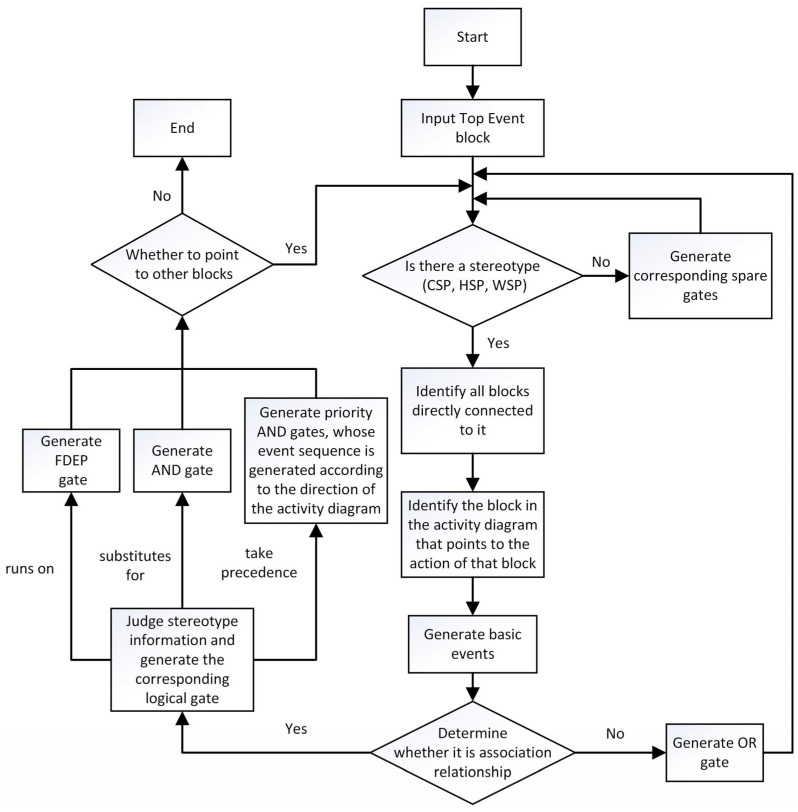
Dynamic fault tree generation steps.

The proposed dynamic fault tree generation method starts from the top event and identifies blocks that are directly associated with the top event block or connected through activity diagram events. If the connection relationship is a composition, an OR gate is created, and the associated block is transformed into a basic event. If the operations of one block in the activity diagram point to the operations of another block, then this block becomes a sub-event of the other block. The stereotype of this block needs to be determined. If the module has spare part construction information, the corresponding spare part gate needs to be generated. Subsequently, the presence of stereotype types between associated modules is examined. If there is a stereotype, generate the corresponding logical gate based on the semantic comparison table. Then, identify all the incentive events that cause its failure. These events are then connected using logic gates and reasoned through layer by layer until SysML model cannot be decomposed any further. The specific algorithm flow is shown in Figure 4.

### 3.4. Quantitative Analysis of Dynamic Fault Tree Based on Model Probability

The dynamically generated fault tree includes two static logical gates, AND gate and OR gate, as well as dynamic logical gates, including Cold Spare Gate, Warm Spare Gate, Hot Spare Gate, Function-Related Gate, and Priority AND gate. In order to perform quantitative analysis of the fault tree, we convert the logical gates in the fault tree into a parametric diagram, which combines input event probabilities in a specified way to determine output event probabilities. If the combination of input events is satisfied, the output event will occur. In order to save space, this article only provides a detailed introduction to a few of the dynamic logic gates.

**Figure 5:**
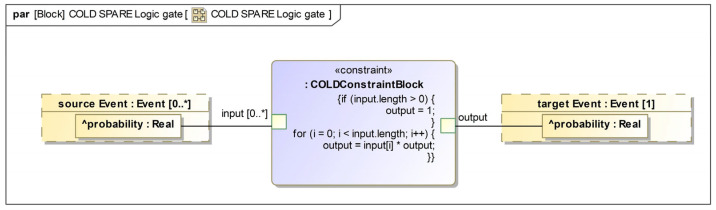
Parametric diagram of dynamic logic gate.

As shown in Figure 5, the Cold Spare Gate is a dynamic logic gate with p3 as its input event and s3 as its output event. However, while p3 is in operation, there is also a standby spare part p3’ in a dormant state. When p3 fails, p3’ will be activated to replace p3 to ensure the normal operation of s3. So, s3 event will only fail when p3’ fails. The function correlation gate is another dynamic logic gate that is used. Its input events are p4, t4, and the output event is s4. Unlike other logic gates, the function correlation gate has a trigger event q4. When the trigger event q4 occurs, the output event s4 will also occur. It is generally assumed that all related events have occurred when the trigger event meets the occurrence conditions.

After categorizing all the events in the previously generated dynamic fault tree into basic events, transitional events, and top events and transforming all the logic gates into SysML parametric diagrams, an internal block diagram can be established based on the previously generated dynamic fault tree. Simulation calculations can be performed in the internal block diagram, and quantitative analysis results can be obtained based on the specification formulas in each logic gate parametric diagram.

## 4. Experimental Case Analysis

### 4.1. FMEA Generation and Risk Assessment

**Figure 6:**
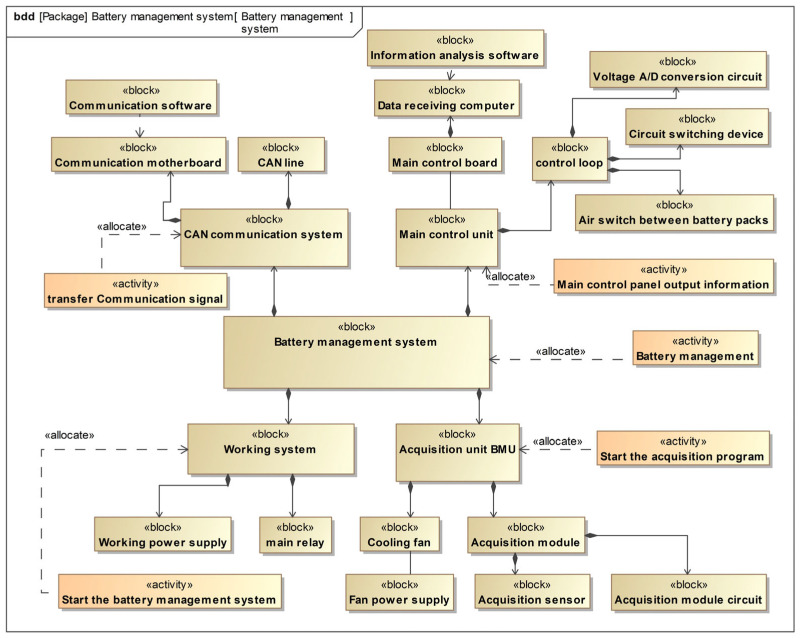
System structure diagram of battery management system.

The power battery management system consists of four main components [36]: the CAN communication system, the working system, the acquisition unit, and the main control unit, as illustrated in Figure 6. The working system supplies power to the battery tube system while the CAN communication system transmits system data. The acquisition unit collects environmental data related to the battery through sensors, and the main control unit is responsible for analyzing this data.

In this stage, the battery management system’s FMEA table was generated using the method proposed in this article, which outlines the functions and potential failure modes of each component. Experts are then tasked with expanding this initial FMEA table to include additional information, such as the severity of failure modes, detectability, incidence, and RPN. Table 2 shows the final FMEA table for the power battery management system. It is important to note that a fault execution degree of 85% is generally considered for battery management systems, and therefore, it is necessary to consider the severity of failure modes when the RPN exceeds 150 [37].

Based on the severity classification of fault modes, line failure and sensor failure in complex environments both reach level 1 hazard degree standards. To improve the system architecture, we will adjust these two components and update the block definition diagram. The new diagram includes two CAN circuits with the same function so that when one fails, the other can replace it. Additionally, the sensors are in the form of cold spare parts to ensure that the system remains operational. The updated system structure will be used to generate a new FMEA analysis and evaluation, and the failure block RPN level will be verified until it meets the predetermined standards.

### 4.2. Automatic Dynamic Fault Tree Generation Process

**Figure 7:**
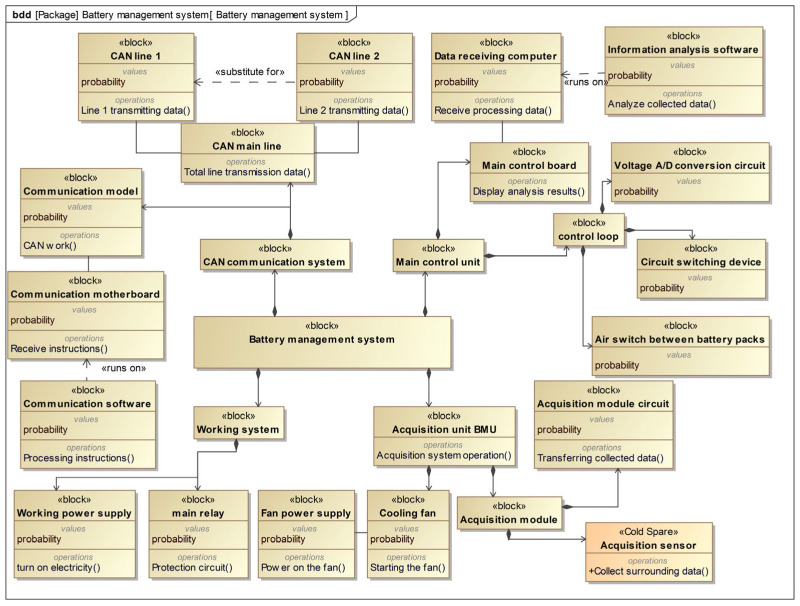
SysML block definition diagram for the battery management system.

**Figure 8:**
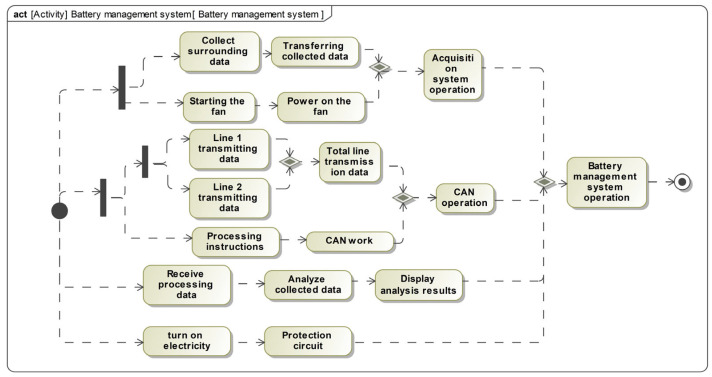
Activity diagram for the battery management system.

Based on the FMEA form and risk assessment conducted by experts, it has been determined that the acquisition sensor fault and CAN line fault have reached level 1 standard. In order to address these issues, we have implemented enhancements for these two components. For acquisition sensors, mainly the IP3281 model AFE acquisition chip, use cold spare parts for redundancy and testing to ensure its compatibility and performance; if the main sensor fails, these cold spare parts will take over the main sensor to continue to work, thus ensuring the continuous and stable operation of the data acquisition system. Additionally, we have added a new CAN communication line, which has the same function as the original line and can serve as a replacement if needed. The updated SysML block definition diagram is presented in Figure 7, while the SysML activity diagram for the power battery system is shown in Figure 8. The activity name in the SysML activity diagram matches the operation attribute name of the block in the SysML block definition diagram, thereby linking the two diagrams when using the algorithm proposed in this paper. The blocks in the SysML block definition diagram represent the base or transition events, while the sequence of activities in the activity diagram represents the transmission of failures.

### 4.3. Quantitative Analysis of Dynamic Fault Tree for Battery Management System

**Figure 9:**
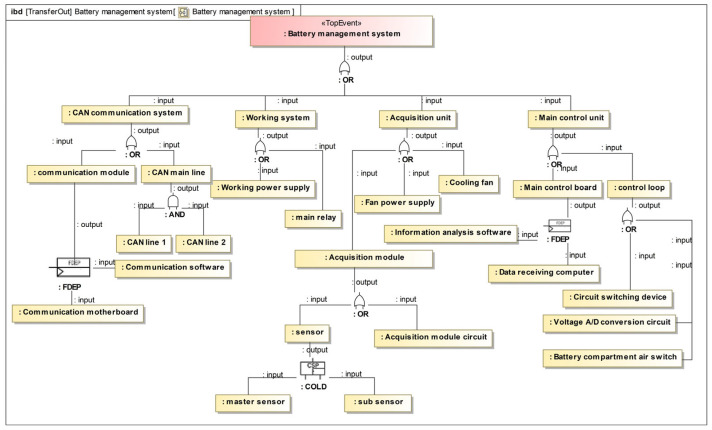
Visualized fault tree for the battery management system.

In the previous section, we completed the generation of a dynamic fault tree. Figure 9 displays all possible combinations of faults in the battery management system. The dynamic fault tree generated in this paper differs slightly from the static fault tree that was manually created. This difference is mainly due to the addition of dynamic logic gates. For example, the failure mode of a cold spare parts door is treated similarly to that of a door. Table 3 shows the occurrence probability of each basic event. By simulating the power battery management system using modeling tools, the failure probability of the top event was obtained as 0.11981. This result is smaller than that obtained by the manually generated fault tree, which demonstrates the advantage of dynamic fault trees over static fault trees. Furthermore, it confirms that the design of the battery management system meets the safety requirements.

## 5. Conclusions

Through the performance evaluation of the design process of the power battery management system, it is found that the failure probability of the top event is 0.11981, and the simulation results are superior to the static fault tree, which verifies that the design of the battery management system meets the safety requirements and proves the feasibility of the method. The main contribution of this paper is to propose a reliability analysis method based on a SysML block definition diagram and an activity diagram. This method cuts the cost and time of safety analysis by automating model analysis, which lowers labor and tool costs and reduces errors during system development. Since the fault transmission information is derived from the system model, the consistency between different security analysis components is enhanced. Compared with traditional methods, the safety analysis results based on a dynamic fault tree can be updated with the changes in system design and can provide a more comprehensive and accurate safety analysis.

Although the method proposed in this paper has achieved remarkable results in the reliability analysis of embedded systems, there are still challenges and future research directions. In the case study in this paper, only spare parts and functionally dependent gates in the dynamic fault tree were considered. Priority and sequentially dependent gates were not converted. Second, security analysis results are also affected by the creation of dynamic fault trees, which require people with a deep understanding of the system. Furthermore, the quantitative analysis in the SysML modeling tool only reduced logic gates, and during conversion, only the determined values of basic event failure probability were considered. No triangular fuzzy operator was used to solve the failure rate. There is still room for improvement in these areas.

## Figures and Tables

**Table 1:** Semantic comparison table between SysML model and dynamic fault tree.

Type of Profile Extension	SysML Model Elements	Fault Tree Elements
No extension	Block composition relation	The basic event of the fault tree OR gate
Extension of temporal characteristics	“runs on”	Function dependent gate
	“take precedence”	Priority AND gate
Expansion of redundancy	“Cold Spare”	Cold spare gate
	“Warm Spare”	Warm spare gate
	“Hot Spare”	Hot spare gate
	“substitutes for”	AND gate

**Table 2:** Failure mode and effects analysis of power battery management system (FMEA).

Component	Function	Failure Mode	Cause	S	O	D	RPN
Working system	Start the battery management system	System not working	No energy supply	7	4	3	84
			Main relay fault	6	3	4	72
		Instruction error	Input instruction error	2	4	6	48
CAN communication system	Communication signal transmission	Signal transmission failure	Communication host fault	7	4	4	112
			CAN line fault	9	5	6	270
			Communication software fault	6	4	6	144
Acquisition unit BMU	Collect surrounding information	Failed to collect surrounding information	No fan power supply	7	4	3	84
			Acquisition fan fault	5	4	4	80
			Acquisition sensor fault	9	8	5	360
		Transmission failure	Collection module circuit fault	5	3	4	60
Main control unit ECU	Analyze collected information	Failure to analyze information	Analysis software runs	6	4	6	144
			Voltage A/D conversion circuit fault	6	4	4	96
			Circuit switching device fault	4	3	3	36
			Control loop failure	6	4	3	72
			Collection information not received	7	3	2	42
		Output information failure	Data receiving computer fault	6	5	4	120

**Table 3:** Failure probability table of equipment.

Basic Event	Failure Probability	Basic Event	Failure Probability
Working power supply	1.8 × 10^−5^	Acquisition module circuit	2.7 × 10^−4^
Main relay	4.96 × 10^−2^	Cooling fan	1.71 × 10^−2^
Communication motherboard	1.75 × 10^−2^	Fan power supply	1.92 × 10^−4^
Communication software	2.5 × 10^−3^	Data receiving computer	3.36 × 10^−4^
CAN line 1	2.3 × 10^−2^	Information analysis software	2.5 × 10^−4^
CAN line 2	2.3 × 10^−2^	Voltage A/D conversion circuit	2.3 × 10^−2^
Acquisition master sensor	1.97 × 10^−2^	Circuit switching device	1.42 × 10^−2^
Acquisition sub-sensor	1.97 × 10^−2^	Battery room air switch	1 × 10^−4^

## Data Availability

Data are contained within the article.
